# The economic and humanistic burden of bipolar disorder in adults in the United States

**DOI:** 10.1186/s12991-023-00440-7

**Published:** 2023-03-24

**Authors:** Carole Dembek, deMauri Mackie, Kushal Modi, Yingying Zhu, Xiaoli Niu, Todd Grinnell

**Affiliations:** 1 Sunovion Pharmaceuticals Inc., Marlborough, MA USA; 2 Cerner Enviza, North Kansas City, MO USA; 3 Center for the Evaluation of Value and Risk in Health, Tufts Medical Center, Boston, MA USA

**Keywords:** Bipolar depression, Direct healthcare costs, Indirect costs, Health-related quality of life, Burden of illness

## Abstract

**Background:**

Bipolar disorder is associated with functional impairment and diminished health-related quality of life (HRQoL). The purpose of this study was to estimate the annual per patient direct healthcare costs, indirect costs, and HRQoL of patients with bipolar disorder by depressive symptom severity and overall compared to the general population in the US.

**Methods:**

This cross-sectional study used self-reported data from the 2020 US National Health and Wellness Survey. Adult respondents who reported bipolar disorder symptoms in the past 12 months and/or a diagnosis of bipolar disorder were identified (bipolar disorder cohort) and were further classified by depressive symptom severity based on Patient Health Questionnaire (PHQ-9) scores (none/mild = 0–9, moderate = 10–14, severe = 15–27). Annualized direct healthcare costs and indirect costs were calculated from 6-month healthcare resource utilization and work productivity, respectively. A general population cohort was constructed using 2:1 propensity score matching. Multivariate regression models of all-cause hospitalizations in the past 6 months, annualized direct healthcare costs, annualized indirect costs, and HRQoL (eg, EuroQol 5-Dimension Health Questionnaire (EQ-5D)) controlled for confounders (demographic and clinical characteristics).

**Results:**

Of 3583 adults meeting pre-specified criteria for bipolar disorder, 1401 (39.1%) reported none/mild, 889 (24.8%) moderate, and 1293 (36.1%) severe depressive symptom severity. Additionally, 3285 (91.7%) were matched to 6570 adults in the general population. Compared to the general population, adjusted mean hospitalizations (0.53 vs. 0.30), annualized per patient direct healthcare costs ($20,846 vs. $11,391), and indirect costs ($14,795 vs. $9274) were significantly greater for the bipolar disorder cohort (all p < 0.001); adjusted HRQoL (EQ-5D: 0.69 vs. 0.79) was significantly worse (p < 0.001). By depressive symptom severity, adjusted mean hospitalizations (none/mild = 0.30, moderate = 0.50, severe = 0.46), direct healthcare costs ($14,389, $22,302, $21,341), and indirect costs ($10,799, $17,109, $18,470) were significantly greater for moderate and severe compared to none/mild depressive symptom severity (all p < 0.01); adjusted HRQoL (EQ-5D: 0.77, 0.67, 0.59) was significantly worse (p < 0.001).

**Conclusions:**

Among respondents with bipolar disorder, those with moderate to severe depression had greater direct healthcare costs and indirect costs as well as worse HRQoL than those with mild or no depressive symptoms. Treatment targeting reduction in depressive symptoms may reduce the economic and humanistic burden of bipolar disorder.

## Introduction

Bipolar disorder is an affective disorder characterized by recurrent manic (bipolar I disorder) or hypomanic (bipolar II disorder) episodes alternating with depressive episodes [1]. The annual prevalence of bipolar disorder is estimated to be 2.8% in the US [2], which may be an underestimation due to under or delayed diagnosis [3]. Symptomatic episodes occur approximately 43–50% of the time [3, 4], and most symptomatic time (70%) is spent in a depressed state (bipolar depression) [4].

The impact of bipolar disorder on patients’ lives is substantial and wide-ranging. Compared to the general population, patients with bipolar disorder have increased risk of cardiovascular disease, obesity, diabetes, hyperglycemia, dyslipidemia, metabolic syndrome, respiratory disease, and migraine headaches [5–7]. In older adults with bipolar disorder, women may be more likely to experience physical comorbidities than men [6]. Co-occurring psychiatric conditions such as substance abuse, anxiety, and borderline personality disorders are also more prevalent in individuals with bipolar disorder compared to the general population [3]. Life expectancy with bipolar disorder is reduced by 9–20 years [8]. Additionally, bipolar disorder has been associated with impaired psychosocial functioning, unemployment, and loss of productivity [5, 9].

Patients with bipolar disorder report worse health-related quality of life (HRQoL) compared to the general population [10, 11]. Bipolar disorder symptoms as well as impaired functioning and productivity in patients with bipolar disorder have been associated with reduced HRQoL [12–14]. Depressive symptoms are more likely associated with worse HRQoL than manic symptoms [13, 15, 16].

The annual economic burden of bipolar disorder in the United States is estimated to be $202 billion [17]. Indirect costs associated with unemployment, productivity loss, and caregiver burden are the main cost driver comprising 72% of total costs [17]. Direct healthcare costs, including inpatient hospitalizations, emergency room visits, and outpatient visits comprise approximately 25% of total costs or $46 billion annually [17]. Direct healthcare costs for individuals with bipolar disorder are estimated to be $25 billion higher than direct healthcare costs for the general population [17].

The economic and humanistic burden of bipolar disorder in the US has not been estimated by severity of depressive symptoms. The aim of this study was to estimate the annual per patient direct healthcare costs and indirect costs as well as the HRQoL of patients with bipolar disorder by depressive symptom severity. Our hypothesis was that economic and humanistic burden would be greater for patients with greater depressive symptom severity. We also report the annual per patient direct healthcare costs, indirect costs, and HRQoL of the general population in the US for comparison.

## Methods

### Data source and study population

Data used in this retrospective, population-based, observational, cross-sectional study are from the 2020 US National Health and Wellness Survey (NHWS), which is a nationally representative database of patient-reported outcomes covering attitudes, behaviors, characteristics, and demographics. Adult respondents were recruited from an existing, general-purpose, web-based consumer panel, with stratified random sampling within the survey panel to ensure representativeness in terms of age and gender. The NHWS is internet-based and self-administered, and data were collected from respondents between April and July 2020. Following the survey logic, not all questions may be presented to all respondents. Respondents were classified as having bipolar disorder or not (general population) based on respondents’ self-reported experience of bipolar disorder in the past 12 months or subject endorsement of a physician diagnosis of bipolar disorder. Respondents with bipolar disorder were further classified by depressive symptom severity using the Patient Health Questionnaire (PHQ-9) (none/mild = 0–9; moderate = 10–14; severe = 15+) [18], a 9-item questionnaire which assesses severity of depressive symptoms over the past 2 weeks.

### Ethical considerations

This study was conducted in accordance with ethical principles consistent with the Declaration of Helsinki and International Conference on Harmonisation (ICH), Good Clinical Practice, Good Pharmacoepidemiology Practice. All respondents explicitly agreed to participate in the NHWS and were provided fair-market value incentives for participation. The 2020 US NHWS was reviewed and approved by the Pearl Institutional Review Board (IRB; Indianapolis, IN, USA). Data were anonymized for use in this study and, as such, did not require further IRB approval.

### Outcomes and other variables

The NHWS collects a wide range of demographic and clinical variables. Demographic characteristics included age, sex, employment status, race/ethnicity, marital status, education level, household income, and health insurance status. Clinical characteristics included body mass index (BMI), smoking status, alcohol use, exercise behavior, Charlson Comorbidity Index (CCI) [19], cardiometabolic comorbidities, PHQ-9, and the 7-item General Anxiety Disorder (GAD-7) scale [20].

For respondents with bipolar disorder, additional data were collected including type of bipolar disorder, age at diagnosis, the type of healthcare practitioner who diagnosed bipolar disorder, whether or not a diagnosis of major depressive disorder (MDD) was made prior to bipolar disorder diagnosis, number of depressive episodes (lasting > 2 weeks) in the past year, number of manic episodes (lasting > 1 week) in the past year, and number of hospitalizations related to mood, emotions, or behavior in the past year.

The primary outcome of interest was the economic and humanistic burden of bipolar disorder measured by HCRU, HRQoL, direct healthcare costs, and indirect costs. Self-reported HCRU included hospitalizations, physician visits, and emergency room (ER) visits in the past 6 months for any medical condition (all-cause). Mental health-related hospitalizations were also reported. Work productivity was assessed using the Work Productivity and Activity Impairment (WPAI) Questionnaire, which is a six-item instrument that asks about impairment due to health in the past week [21]. All respondents completed the WPAI item for activity impairment (percentage of impairment in daily activities). Respondents who self-reported being part of the labor force (full-time, part-time, or self-employed) also completed the items for absenteeism (percentage of work time missed) and presenteeism (percentage of impairment experienced while at work).

Two HRQoL instruments were administered. The five-level EuroQol 5-Dimension Health Questionnaire (EQ-5D) is a self-reported measure of health comprised of 5 dimensions: mobility, self-care, usual activities, pain/discomfort, and anxiety/depression [22]. The EQ-5D Index Score is a summary across the 5 domains with a range from 0–1 with a lower score indicating greater disability. The EQ-5D visual analog scale (VAS) asks respondents to indicate their self-rated health from 0–100 with 0 = ‘worst imaginable health state’ and 100 = ‘best imaginable health state’. The revised Medical Outcomes Study 36-Item Short Form Survey Instrument (SF-36) is a multipurpose, generic HRQoL instrument composed of 36 questions, which uses norm-based scoring to allow for comparisons with the general population [23]. The normed physical component summary (PCS) and mental component summary (MCS) scores were reported separately. Health utility scores were estimated from six domains of the SF-36 (SF-6D).

Direct healthcare costs were calculated using self-reported HCRU (hospitalizations, ER visits, and physician visits) multiplied by the age-stratified average cost per visit from the 2018 Medical Expenditure Panel Survey (MEPS), which was the most recently available at the time of the analysis. MEPS-based average costs were not specific to practitioner type or reason for the visit. Direct healthcare costs were annualized by multiplying the 6-month HCRU by two before applying the average cost per visit. Indirect costs were calculated based on the human capital approach. Indirect costs were calculated using hours missed from work in the last 7 days due to health and hours worked in the last 7 days while impaired by health and were multiplied by the average 2019 wage per day by sex and age from the Bureau of Labor Statistics [24]. Indirect costs were annualized based on 50 paid working weeks per year. Costs were reported in 2019 USD.

### Statistical analysis

Demographic, health, and bipolar disorder-specific characteristics were reported using means and standard deviations for continuous variables and counts and proportions for categorical variables. Multivariate regression models were used to compare hospitalizations, direct healthcare costs, indirect costs, and HRQoL. Generalized linear models (GLMs) were estimated using a negative binomial distribution for hospitalizations, a gamma distribution for direct healthcare costs and indirect costs, and a normal distribution for HRQoL. Covariates were included to control for confounders and were selected based on review of the literature or bivariate significance (p-value < 0.05). Multicollinearity was evaluated in all models. Models of HRQoL and direct healthcare costs by depressive symptom severity controlled for age, sex, race, employment, health insurance, smoking status, alcohol use, exercise behavior, BMI, education level, income level, and CCI. The model of indirect costs by depressive symptom severity used the same control variables except for employment. Frequency of manic episodes was not included in the models. Propensity score matching was used to construct a general population cohort. A 2:1 match was used to identify two members of the general population without bipolar disorder for each respondent with bipolar disorder. The matching model controlled for age, sex, race, employment, marital status, health insurance, smoking status, alcohol use, exercise behavior, BMI, education level, income level, and CCI. Respondents with bipolar disorder who were unable to be matched to the general population were dropped. Models comparing respondents with bipolar disorder vs. the general population controlled for CCI. Adjusted means and 95% confidence intervals (CIs) were calculated using the fitted GLMs. Descriptive statistics and bivariate analyses were conducted using SPSS v28 (IBM Corp, New York, USA), and regression models were built in SAS v9.4 (SAS Corporation, North Carolina, USA). Statistical significance was defined as *p*-value < 0.05.

## Results

### Respondent characteristics

**Fig. 1:**
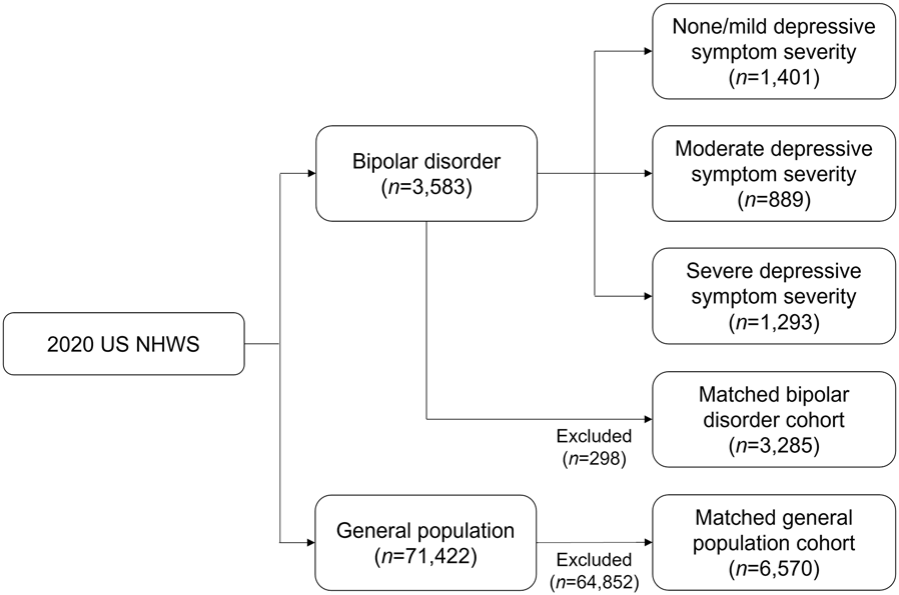
Flow diagram. *n*, number of respondents

There were 3583 respondents meeting pre-specified criteria for bipolar disorder in the 2020 US NHWS (Fig. 1). Of those respondents, 1401 (39.1%) reported none/mild, 889 (24.8%) moderate, and 1293 (36.1%) severe depressive symptom severity. Among respondents with bipolar disorder, 3285 (91.7%) were matched to the general population (*n* = 6570). The demographic and clinical characteristics of the bipolar disorder and general population cohorts were similar except for greater comorbidities among respondents with bipolar disorder (CCI = 0.7 vs. 0.6, *p* = 0.001) (Table 1).

**Table 1:** Demographic and clinical characteristics

Characteristic	Respondents with bipolar disorder by depressive symptom severity			Respondents with bipolar disorder (*n* = 3285)	General population (n = 6570)
	None/mild (*n* = 1401)	Moderate (*n* = 889)	Severe (*n* = 1293)		
Age, mean (SD)	38.8 (14.5)	33.0 (12.2)^a^	34.4 (11.5)^a^	36.3 (13.3)	35.9 (14.7)
Female, n (%)	833 (59.5%)	513 (57.7%)^a^	848 (65.6%)^a^	2031 (61.8%)	4046 (61.6%)
Race/ethnicity, n (%)					
White	796 (56.8%)	439 (49.4%)^a^	738 (57.1%)^a^	1828 (55.6%)	3585 (54.6%)
Black	203 (14.5%)	132 (14.8%)^a^	134 (10.4%)^a^	433 (13.2%)	878 (13.4%)
Hispanic	273 (19.5%)	208 (23.4%)^a^	310 (24.0%)^a^	700 (21.3%)	1443 (22.0%)
Other	129 (9.2%)	110 (12.4%)^a^	111 (8.6%)^a^	324 (9.9%)	664 (10.1%)
Region, n (%)					
Northeast	241 (17.2%)	164 (18.4%)	203 (15.7%)	556 (16.9%)	1171 (17.8%)
Midwest	283 (20.2%)	169 (19.0%)	234 (18.1%)	629 (19.1%)	1341 (20.4%)
South	577 (41.2%)	393 (44.2%)	564 (43.6%)	1390 (42.3%)	2629 (40.0%)
West	300 (21.4%)	163 (18.3%)	292 (22.6%)	710 (21.6%)	1429 (21.8%)
Marital status, n (%)					
Single	808 (57.7%)	521 (58.6%)	730 (56.5%)	1872 (57.0%)	3809 (58.0%)
Married/living with Partner	593 (42.3%)	368 (41.4%)	563 (43.5%)	1413 (43.0%)	2761 (42.0%)
Education, n (%)					
Less than college/university	1012 (72.2%)	643 (72.3%)^a^	988 (76.4%)^a^	2405 (73.2%)	4832 (73.5%)
College/university degree or higher	389 (27.8%)	246 (27.7%)^a^	305 (23.6%)^a^	880 (26.8%)	1738 (26.5%)
Any employment (full-time/part-time/self-employed), n (%)	721 (51.5%)	517 (58.2%)^a^	647 (50.0%)^a^	1743 (53.1%)	3506 (53.4%)
Any health insurance, n (%)	1164 (83.1%)	719 (80.9%)^a^	1008 (78.0%)^a^	2672 (81.3%)	5308 (80.8%)
Annual household income, n (%)					
Less than $50,000	880 (62.8%)	521 (58.6%)^a^	854 (66.0%)^a^	2055 (62.6%)	4227 (64.3%)
$50,000 or greater	521 (37.2%)	368 (41.4%)^a^	439 (34.0%)^a^	1230 (37.4%)	2343 (35.7%)
Smoking status, n (%)					
Never smoked	559 (39.9%)	383 (43.1%)^a^	411 (31.8%)^a^	1274 (38.8%)	2507 (38.2%)
Former smoker	332 (23.7%)	178 (20.0%)^a^	262 (20.3%)^a^	736 (22.4%)	1518 (23.1%)
Current smoker	510 (36.4%)	328 (36.9%)^a^	620 (48.0%)^a^	1275 (38.8%)	2545 (38.7%)
Any alcohol use, n (%)	884 (63.1%)	649 (73.0%)^a^	887 (68.6%)^a^	2197 (66.9%)	4388 (66.8%)
Any exercise in the past month, n (%)	909 (64.9%)	607 (68.3%)^a^	764 (59.1%)^a^	2103 (64.0%)	4240 (64.5%)
BMI, kg/m^2^, mean (SD)	29.4 (8.0)	28.7 (8.4)	28.9 (8.5)	28.9 (8.3)	28.9 (8.2)
CCI, mean (SD)	0.7 (2.0)	0.8 (1.6) ^a^	1.1 (2.3) ^a^	0.7 (1.4)	0.6 (1.4)^b^
Cardiometabolic comorbidities, n (%)					
Obesity	566 (39.7%)	303 (34.1%)^a^	486 (37.6%)^a^	1273 (38.8%)	2400 (36.5%)^b^
Hypertension	309 (22.1%)	160 (18.0%)^a^	308 (23.8%)^a^	721 (21.9%)	1141 (17.4%)^b^
Migraine	270 (19.3%)	207 (23.3%)^a^	417 (32.3%)^a^	827 (25.2%)	940 (14.3%)^b^
Diabetes	176 (12.6%)	104 (11.7%)	147 (11.4%)	374 (11.4%)	623 (9.5%)
Cardiovascular disease	123 (8.8%)	81 (9.1%)	145 (11.2%)	288 (8.8%)	431 (6.6%)^b^
Cerebrovascular disease	47 (3.4%)	32 (3.6%)	63 (4.9%)	111 (3.4%)	149 (2.3%)^b^
PHQ-9, mean (SD)	4.9 (3.3)	12.1 (1.4)^a^	19.9 (3.9)^a^	12.0 (7.2)	7.6 (7.0)^b^
GAD-7, mean (SD)	5.7 (4.8)	10.1 (4.1)^a^	14.5 (4.9)^a^	9.9 (6.1)	6.1 (5.8)^b^

BMI, body mass index; CCI, Charlson Comorbidity Index; GAD-7, 7-item General Anxiety Disorder scale; *n*, number of respondents; PHQ-9, Patient Health Questionnaire; SD, standard deviation

^a^Significant at *p* < 0.05 across depressive symptom severity cohorts [reference = none/mild]

^b^Significant at *p* < 0.05 for bipolar disorder vs. general population [reference]

Across the current depressive symptom severity cohorts, demographic and clinical characteristics were significantly different (Table 1). Respondents with none/mild depressive symptom severity were the oldest on average (none/mild = 38.8 years, moderate = 33.0 years, severe = 34.4 years; *p* < 0.001) and had the lowest CCI score (0.7, 0.8, 1.1; *p* < 0.001). Respondents with moderate depressive symptom severity were most likely to be male (none/mild = 40.5%, moderate = 42.3%, severe = 34.4%; *p* < 0.001) and employed (51.5%, 58.2%, 50.0%; *p* = 0.001) and to have an annual household income of $50,000 or greater (37.2%, 41.4%, 34.0%; *p* = 0.002). Respondents with moderate depressive symptom severity also had the greatest rates of never smoked (39.9%, 43.1%, 31.8%; *p* < 0.001), alcohol use (63.1%, 73.0%, 68.6%; *p* < 0.001), and exercise in the past month (64.9%, 68.3%, 59.1%; *p* < 0.001). Respondents with severe depressive symptom severity were most likely to have less than a college or university degree (72.2%, 72.3%, 76.4%; *p* = 0.026) and had the greatest rates of being a current smoker (36.4%, 36.9%, 48.0%; *p* < 0.001).

Approximately 40% of respondents in the bipolar disorder cohort reported a current diagnosis or presence of symptoms consistent with bipolar I disorder, 40% with bipolar II disorder, and 20% with unspecified bipolar disorder (*p* = 0.134) (Table 2). The average numbers of depressive (none/mild = 3.1, moderate = 4.5, severe = 7.4; *p* < 0.001) and manic (2.6, 3.7, 5.3; *p* < 0.001) episodes were twice as high for respondents with current severe compared to none/mild depressive symptom severity.

**Table 2:** Additional characteristics of respondents with bipolar disorder

Characteristic	Respondents with bipolar disorder by depressive symptom severity		
	None/mild (*n* = 1401)	Moderate (*n* = 889)	Severe (*n* = 1293)
Number of depressive episodes in the past year, mean (SD)	3.1 (5.0)	4.5 (5.3)^b^	7.4 (7.3)^b^
Number of manic episodes in the past year, mean (SD)	2.6 (4.6)	3.7 (5.1)^b^	5.3 (6.2)^b^
Number of hospitalizations related to mood, emotions, or behavior in the past year, mean (SD)	0.7 (3.9)	1.5 (5.2)^b^	1.5 (6.0)^b^
Type of bipolar disorder, n (%)^a^			
Type I	394 (40.7%)	184 (37.0%)	334 (40.5%)
Type II	365 (37.7%)	222 (44.7%)	322 (39.1%)
Unspecified	208 (21.5%)	91 (18.3%)	168 (20.4%)
Age at diagnosis of bipolar disorder, mean (SD)^a^	27.3 (12.6)	24.8 (12.2)^b^	24.9 (11.5)^b^
Healthcare practitioner who diagnosed bipolar disorder, n (%)^a^			
Psychiatrist	634 (65.6%)	283 (56.9%)	473 (57.4%)
Primary Care Physician/General Practitioner/Internist	143 (14.8%)	86 (17.3%)	137 (16.6%)
Psychologist	116 (12.0%)	81 (16.3%)	137 (16.6%)
Nurse Practitioner/Physician Assistant in a psychiatry practice	29 (3.0%)	18 (3.6%)	27 (3.3%)
Nurse Practitioner/Physician Assistant in a primary care physician/general practitioner/internist practice	16 (1.7%)	11 (2.2%)	17 (2.1%)
Other	29 (3.0%)	18 (3.6%)	33 (4.0%)
Diagnosis of MDD prior to bipolar disorder diagnosis, n (%)^a^	582 (60.2%)	361 (72.6%)^b^	636 (77.2%)^b^

MDD, major depressive disorder; *n*, number of respondents; SD, standard deviation

^a^Number of respondents was lower for type of bipolar disorder, age at diagnosis of bipolar disorder, healthcare practitioner who diagnosed bipolar disorder, and diagnosis of MDD prior to bipolar disorder diagnosis (none/mild: *n* = 967, moderate: *n* = 497, severe: *n* = 824)

^b^Significant at *p* < 0.05 across depressive symptom severity cohorts [reference = none/mild]

### Economic burden in respondents with bipolar disorder vs. general population

After adjusting for demographic and clinical characteristics, respondents in the bipolar disorder cohort reported an average of 0.53 (95% CI 0.43, 0.66) all-cause hospitalizations in the past 6 months compared to 0.30 (0.26, 0.35) for the matched general population (Table 3). Average annualized direct healthcare costs were $20,846 ($17,654, $24,615) in the bipolar cohort compared to $11,391 ($10,129, $12,811) in the matched general population. Average annualized indirect costs were $14,795 ($13,867, $15,786) in the bipolar cohort compared to $9274 ($8861, $9705) in the matched general population.

**Table 3:** Adjusted hospitalizations, direct healthcare costs, and indirect costs

Outcome	Respondents with bipolar disorder by depressive symptom severity			Respondents with bipolar disorder (*n* = 3285)	General population (*n* = 6570)
	None/mild (*n* = 1401)	Moderate (*n* = 889)	Severe (*n* = 1293)		
All-cause hospitalizations in the past 6 months, mean (95% CI)	0.30 (0.24, 0.37)	0.50 (0.39, 0.64)	0.46 (0.38, 0.57)	0.53 (0.43, 0.66)	0.30 (0.26, 0.35)
Annualized total direct healthcare costs, per patient, mean (95% CI)	$14,389 ($12,390, $16,711)	$22,302 ($18,420, $27,001)	$21,341 ($18,231, $24,981)	$20,846 ($17,654, $24,615)	$11,391 ($10,129, $12,811)
Annualized indirect costs, per patient, mean (95% CI)	$10,799 ($9938, $11,734)	$17,109 ($15,487, $18,901)	$18,470 ($16,889, $20,200)	$14,795 ($13,867, $15,786)	$9274 ($8861, $9705)

BMI, body mass index; CCI, Charlson Comorbidity Index; CI, confidence interval; *n*, number of respondents

Models by depressive symptom severity adjusted for age, sex, race, employment (models of hospitalizations and directs costs only), health insurance, smoking status, alcohol use, exercise behavior, BMI, education level, income level, and CCI. Models comparing respondents with bipolar disorder vs. matched general population adjusted for CCI

### Economic burden by depressive symptom severity

**Fig. 2:**
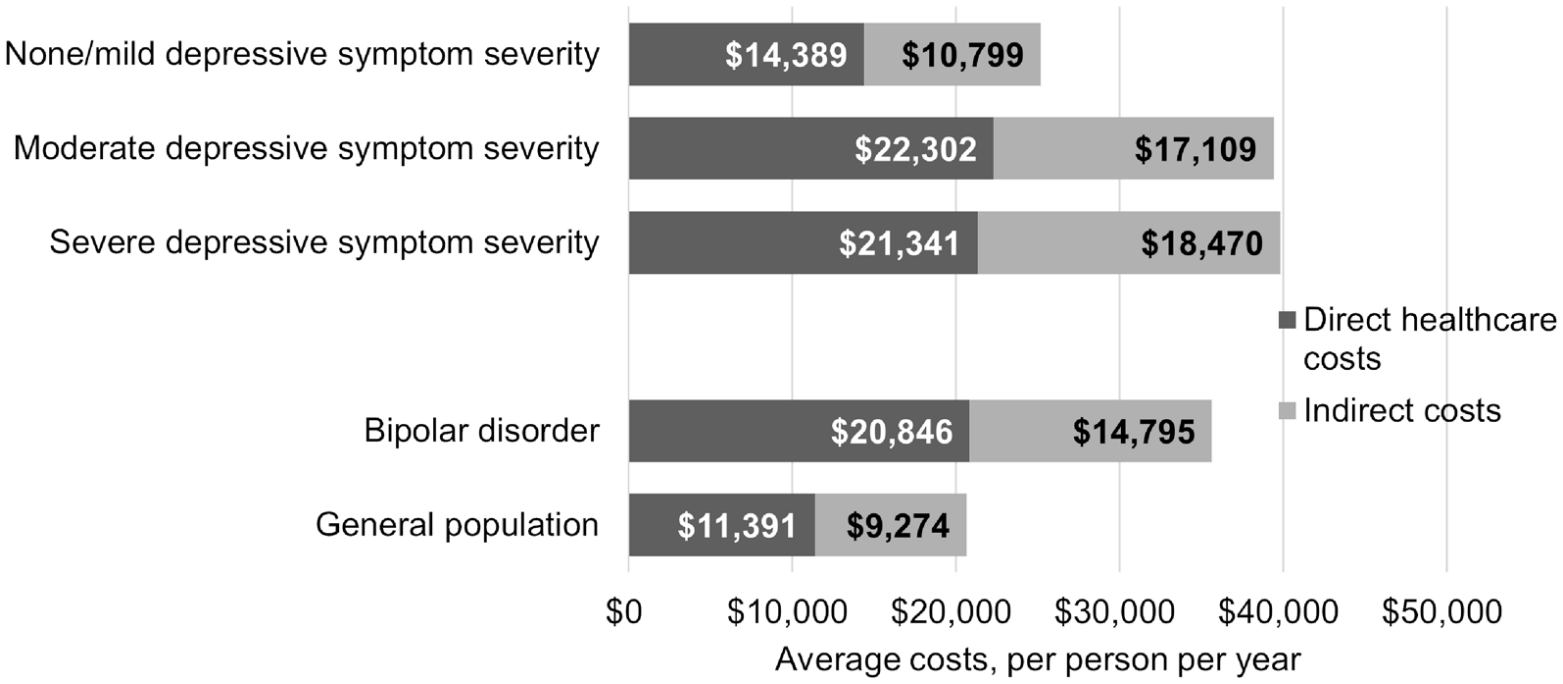
Adjusted direct healthcare costs and indirect costs. BMI, body mass index; CCI, Charlson Comorbidity Index. Models by depressive symptom severity adjusted for age, sex, race, employment (model of direct healthcare costs only), health insurance, smoking status, alcohol use, exercise behavior, BMI, education level, income level, and CCI. Models comparing respondents with bipolar disorder vs. matched general population adjusted for CCI

After adjusting for demographic and clinical characteristics, all-cause hospitalizations in the past 6 months (moderate: mean (95% CI) 0.50 (0.39, 0.64); severe: 0.46 (0.38, 0.57)), annualized direct healthcare costs [$22,302 ($18,420, $27,001); $21,341 ($18,231, $24,981)), and annualized indirect costs ($17,109 ($15,487, $18,901); $18,470 ($16,889, $20,200)] were not significantly different when comparing respondents with moderate or severe depressive symptom severity. However, both moderate and severe depressive symptom cohorts had significantly greater economic burden compared to the none/mild cohort [hospitalizations: 0.30 (0.24, 0.37), direct healthcare costs: $14,389 ($12,390, $16,711), indirect costs: $10,799 ($9938, $11,734)]. Combined average direct healthcare costs and indirect costs were similar for respondents with current moderate and severe depressive symptom severity (Fig. 2). Combined average direct healthcare costs and indirect costs were greater among respondents with bipolar disorder compared to the general population cohort.

### Humanistic burden in respondents with bipolar disorder vs. general population

After adjusting for demographic and clinical characteristics, the average EQ-5D score in respondents in the bipolar disorder cohort was 0.69 (95% CI 0.68, 0.69) compared to 0.79 (0.79, 0.80) for the matched general population (Table 4). Both SF-36 components were worse for respondents in the bipolar disorder cohort (MCS: mean (95% CI) 35.4 (35.0, 35.8); PCS: 46.5 (46.2, 46.9) compared to the matched general population (MCS: 42.0 (41.7, 42.3); PCS: 49.6 (49.3, 49.8)).

**Table 4:** Adjusted HRQoL

Outcome	Respondents with bipolar disorder by depressive symptom severity			Respondents with bipolar disorder (*n* = 3285)	General population (*n* = 6570)
	None/mild (*n* = 1401)	Moderate (*n* = 889)	Severe (*n* = 1293)		
EQ-5D, mean (95% CI)	0.77 (0.76, 0.78)	0.67 (0.66, 0.68)	0.59 (0.58, 0.60)	0.69 (0.68, 0.69)	0.79 (0.79, 0.80)
EQ-5D VAS, mean (95% CI)	70.9 (69.6, 72.3)	62.1 (60.3, 63.8)	56.1 (54.6, 57.5)	63.8 (63.0, 64.7)	71.7 (71.1, 72.3)
SF-36 MCS, mean (95% CI)	42.0 (41.5, 42.5)	35.1 (34.5, 35.8)	28.1 (27.6, 28.6)	35.4 (35.0, 35.8)	42.0 (41.7, 42.3)
SF-36 PCS, mean (95% CI)	48.7 (48.2, 49.2)	44.9 (44.3, 45.6)	44.4 (43.9, 45.0)	46.5 (46.2, 46.9)	49.6 (49.3, 49.8)
SF-6D, mean (95% CI)	0.65 (0.65, 0.66)	0.57 (0.57, 0.58)	0.53 (0.52, 0.54)	0.59 (0.59, 0.60)	0.67 (0.66, 0.67)

BMI, body mass index; CCI, Charlson Comorbidity Index; CI, confidence interval; EQ-5D, EuroQol 5-Dimension Health Questionnaire; MCS, mental component summary; *n*, number of respondents; PCS, physical component summary; SF-36, Medical Outcomes Study 36-Item Short Form Survey Instrument; VAS, visual analog scale

Models by depressive symptom severity adjusted for age, sex, race, employment, health insurance, smoking status, alcohol use, exercise behavior, BMI, education level, income level, and CCI. Models comparing respondents with bipolar disorder vs. matched general population adjusted for CCI

### Humanistic burden by depressive symptom severity

**Fig. 3:**
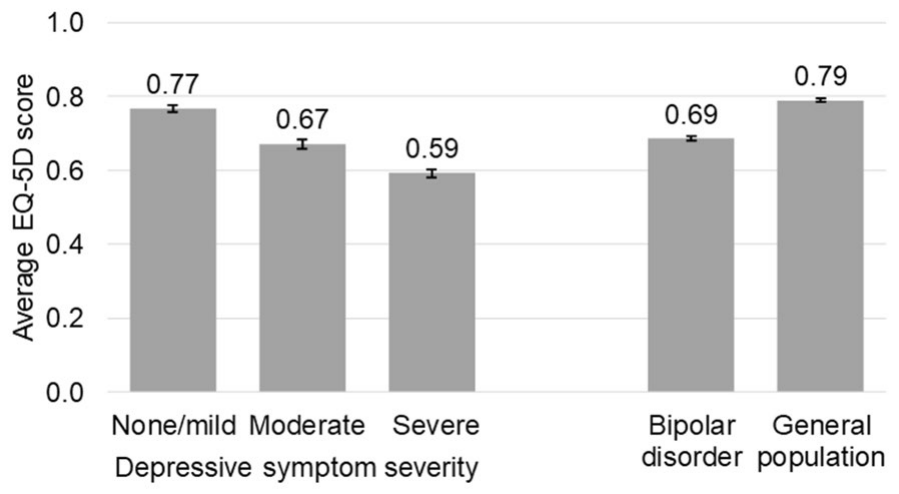
Adjusted HRQoL. BMI, body mass index; CCI, Charlson Comorbidity Index; CI, confidence interval; EQ-5D, EuroQol 5-Dimension Health Questionnaire. Model by depressive symptom severity adjusted for age, sex, race, employment, health insurance, smoking status, alcohol use, exercise behavior, BMI, education level, income level, and CCI. Model comparing respondents with bipolar disorder vs. matched general population adjusted for CCI. Error bars represent 95% CI

After adjusting for demographic and clinical characteristics, the average EQ-5D score was 0.59 (95% CI 0.58, 0.60) for respondents with current severe depressive symptom severity compared to 0.67 (0.66, 0.68) and 0.77 (0.76, 0.78) for moderate and none/mild, respectively. The SF-36 MCS was 28.1 (27.6, 28.6) for respondents with severe depressive symptom severity compared to 35.1 (34.5, 35.8) and 42.0 (41.5, 42.5) for moderate and none/mild, respectively. The SF-36 PCS was 44.9 (44.3, 45.6) and 44.4 (43.9, 45.0) for moderate and severe depressive symptom severity, respectively, which was significantly lower than 48.7 (48.2, 49.2) in the none/mild cohort. EQ-5D scores for respondents with none/mild depressive symptom severity were slightly lower than for the general population (Fig. 3).

## Discussion

Among respondents with bipolar disorder, current moderate or severe depressive symptom severity was associated with greater hospitalizations, direct healthcare costs, and indirect costs compared to none/mild depressive symptom severity. HRQoL was significantly worse with greater levels of depressive symptom severity. To our knowledge, this is the first study comparing differences in economic and humanistic burden of bipolar disorder by depressive symptom severity in a US nationally representative sample. Additionally, respondents with bipolar disorder reported significantly greater hospitalizations, direct healthcare costs, and indirect costs as well as worse HRQoL compared to a matched sample of the general population without bipolar disorder.

Previous estimates of per patient annual all-cause healthcare costs for patients with bipolar disorder ranged from $11,051 to $46,971 (2018 USD) with mental health-related costs accounting for approximately half of direct medical costs (range from literature = $6374–$21,523, 2018 USD) [25]. The current study is aligned with those findings with per patient annual direct healthcare costs of $20,846. Additionally, results from this study suggest that direct healthcare costs are likely driven by patients with moderate or severe depressive symptoms. Annualized all-cause direct healthcare costs were 48–55% greater for respondents with moderate or severe compared to none/mild depressive symptom severity. Slightly greater adjusted direct healthcare costs for moderate compared to severe depressive symptom severity were likely due to greater non-mental health-related hospitalizations as mental health-related hospitalizations were similar in the two cohorts (Table 2).

Multiple studies have demonstrated that depressive symptoms in patients with bipolar disorder are associated with worse work productivity, employment outcomes, and occupational functioning [14, 26, 27]. A recent post-hoc analysis of clinical trial data in patients with bipolar depression estimated annualized indirect costs due to productivity loss of $58,075–$61,235 [28]. In the current study, respondents with severe depressive symptoms (i.e., most comparable to a bipolar depression clinical trial cohort) had average indirect costs of $18,470. The difference in estimates could be due to the study population, the instrument used to measure productivity, or the methods used to estimate indirect costs. However, the current study also suggests that greater levels of depressive symptom severity may be associated with worse work outcomes; annualized indirect costs were 58–71% greater for respondents with moderate or severe compared to none/mild depressive symptom severity.

Many instruments have been used to measure HRQoL in patients with bipolar disorder [10]. EQ-5D utility scores are useful for incorporating humanistic burden in health economic assessments, and SF-36 MCS and PCS scores demonstrate the association of a condition with different aspects (mental and physical) of HRQoL. This study demonstrated that worse HRQoL in patients with bipolar disorder may be driven by moderate or severe depressive symptoms. The association was greater for mental vs. physical health-related HRQoL as measured by the SF-36 MCS and PCS scores, respectively.

In the real world, patients in the euthymic phase of illness may continue to experience worse functioning [29], sleep disturbances [30], and worse HRQoL [31] compared to the general population. Continued impacts on functioning have been associated with residual depressive symptoms [32, 33]. Although statistical significance was not tested directly for depressive symptom severity groups compared to the general population, respondents with current none/mild depressive symptom severity in this study reported greater direct healthcare costs and indirect costs and worse HRQoL compared to the general population.

The association of depressive symptom severity with worse HRQoL and higher costs underscores the importance of identifying, treating, and monitoring depressive symptoms of bipolar disorder in the clinical setting. PHQ-9 has been recommended to assess depressive symptom severity and monitor response to treatment [7]. FDA-approved treatments for acute depressive episodes associated with bipolar disorder include several second-generation antipsychotics: cariprazine, lurasidone, olanzapine-fluoxetine, lumateperone, and quetiapine [5]. While these therapies are largely successful in managing depressive symptoms, they are associated to varying degrees with side effects including weight gain, metabolic syndrome, movement disorders, and sedation [5]. Further innovation in the treatment of bipolar depression is needed to provide more clinical options for specialists and primary care practitioners caring for patients with bipolar disorder.

There were several limitations to this study. First, the diagnosis of bipolar disorder was self-reported by survey respondents and not confirmed with a physician diagnosis. The higher prevalence of bipolar disorder in this study, almost 5% vs 2.8% in other US estimates [2], suggests our sample may over-represent the bipolar disorder population in the US. Second, the outcomes of interest in this study were based on self-reported data and may be subject to recall bias. Respondents were asked to recall the past 6 months of HCRU and the past week of work productivity and HRQoL. Third, the annualization of HCRU and work productivity measures assumes that the incidence and duration of manic and depressive episodes are uniformly consistent across 12 months. Fourth, the analysis by depressive symptom severity did not control for the frequency of manic episodes in the past year, which may also be associated with hospitalizations. A detailed evaluation of manic symptom severity was not collected in the survey. Finally, this analysis was not a comprehensive study of the burden of bipolar disorder. Indirect costs related to unemployment and productivity loss for patients with bipolar disorder have been estimated to be approximately 57% of total indirect costs attributed to bipolar disorder [25]. Additional contributors to indirect costs include premature mortality, productivity loss of caregivers, and healthcare costs of caregivers [25]. However, premature mortality and caregiver information were not collected in the survey.

## Conclusions

Current moderate to severe depressive symptoms were associated with greater direct healthcare costs and indirect costs as well as worse HRQoL for respondents with bipolar disorder. New therapies for the treatment of depressive symptoms may decrease the economic and humanistic burden of bipolar disorder.

## Data Availability

This study used data from the 2020 US NHWS, which are not publicly available.

## Abbreviations

BMI: Body mass index
CCI: Charlson Comorbidity Index
CI: Confidence intervals
EQ-5D: EuroQol 5-Dimension Health Questionnaire
ER: Emergency room
GAD-7: General Anxiety Disorder 7-item scale
GLM: Generalized linear model
HCRU: Healthcare resource utilization
HRQoL: Health-related quality of life
ICH: International Conference on Harmonisation
IRB: Institutional Review Board
MCS: Mental component summary
MDD: Major depressive disorder
MEPS: Medical Expenditure Panel Survey
NHWS: National Health and Wellness Survey
PCS: Physical component summary
PHQ-9: Patient Health Questionnaire 9-item scale
SF-36: 36-Item Short Form Survey Instrument
SF-6D: Six domains of the SF-36
US: United States
USD: United States Dollars
VAS: Visual analog scale
WPAI: Work Productivity and Activity Impairment Questionnaire
